# Small bowel entrapment as a rare complication of lumbar micro discectomy: Case report and literature review

**DOI:** 10.1016/j.ijscr.2025.111234

**Published:** 2025-03-29

**Authors:** Hiba Ben Hassine, Faiez Boughanmi, Mohamed Ridha Zayati, Leith limayem, Ibtissem korbi, Faouzi noomen

**Affiliations:** Department of Visceral Surgery, Fattouma Bourguiba Hospital, Monastir, Tunisia

**Keywords:** Small bowel entrapment, Discectomy, Visceral injury, Case report

## Abstract

**Introduction:**

Laminectomy with lumbar disc excision remains the standard surgical approach for the management of lumbar disc herniation. While the incidence and nature of common complications associated with this procedure are well-documented, ventral perforation leading to intestinal injury is an exceptionally rare occurrence. However, when it does occur, it can result in severe and potentially life-threatening consequences.

**Case presentation:**

We present the case of a 47-year-old male who developed an acute abdomen on the second postoperative day following an L5-S1 discectomy. Exploratory laparotomy revealed small bowel entrapment. Surgical management included segmental resection of the affected bowel segment with the creation of a double ileostomy.

**Discussion:**

Although exceedingly rare, intestinal injuries secondary to ventral perforation during lumbar discectomy can have serious repercussions. Adequate preoperative surgical planning, careful selection of the surgical approach, and meticulous dissection are critical in minimizing the risk of ventral perforation and subsequent bowel injury.

**Conclusion:**

Intestinal injury should be recognized as a potential but uncommon complication of lumbar disc surgery, particularly in patients presenting with acute abdominal symptoms or persistent wound infections postoperatively. Prompt diagnosis and timely surgical intervention are crucial in preventing severe morbidity and mortality.

## Highlights

• Bowel injury after lumbar discectomy is rare but can be fatal if unrecognized.
• Early signs include acute abdomen or prolonged wound infection.
• Imaging plays a crucial role in diagnostic
• Early recognition and timely surgical intervention are essential.
• Prevention is Crucial

## 1 Introduction

Laminectomy with lumbar disc excision remains the standard surgical approach for treating lumbar disc herniation. While the common complications associated with this procedure are well-documented, isolated small bowel entrapment following lumbar discectomy is an exceedingly rare but serious complication, carrying a high risk of morbidity and mortality. A total of 24 publications have reported 23 cases of bowel injury related to this condition [1,2]. This case report follows the 2023 SCARE guidelines [3].

## 2 Case presentation

A 47-year-old male with a history of prior lumbar laminectomy presented with persistent lower back pain radiating down the leg to the ankle. Conservative management, including activity modification and analgesic therapy, failed to alleviate his symptoms. Consequently, he underwent an L5-S1 laminectomy with lumbar disc excision. The immediate postoperative course was uneventful, and he was discharged the following day.

Two days postoperatively, the patient developed abdominal pain, nausea, and vomiting. Clinical examination revealed signs of an acute abdomen, including diminished bowel sounds, abdominal guarding, and rebound tenderness. His vital signs included a blood pressure of 120/70 mmHg, a heart rate of 85 beats per minute, and a temperature of 38.5 °C. Laboratory investigations showed leukocytosis (24,000/mm^3^) and an elevated C-reactive protein level (230 mg/L).

**Fig. 1:**
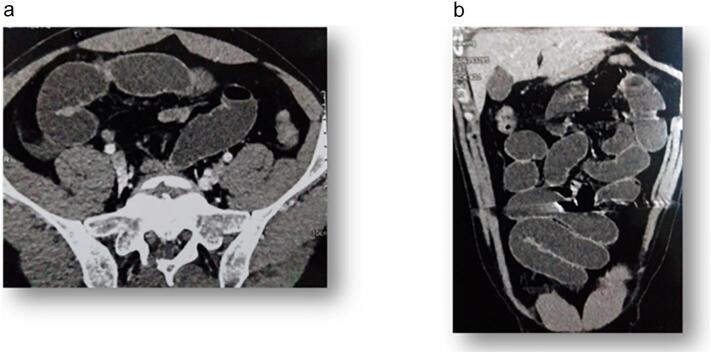
a: CT scan: diffuse dilatation of the small bowel loops with a collapsed colon lumen. b: CT scan: the transition zone appeared to be at the distal ileum regarding the paravertebral space of L5-S1.

Abdominopelvic computed tomography (CT) revealed diffuse small bowel dilatation with a collapsed colon, suggesting a transition point at the distal ileum adjacent to the L5-S1 paravertebral region. Additionally, localized fat stranding and small volumes of extraluminal free fluid were observed (Fig. 1). There was no evidence of pneumoperitoneum or bowel ischemia.

**Fig. 2:**
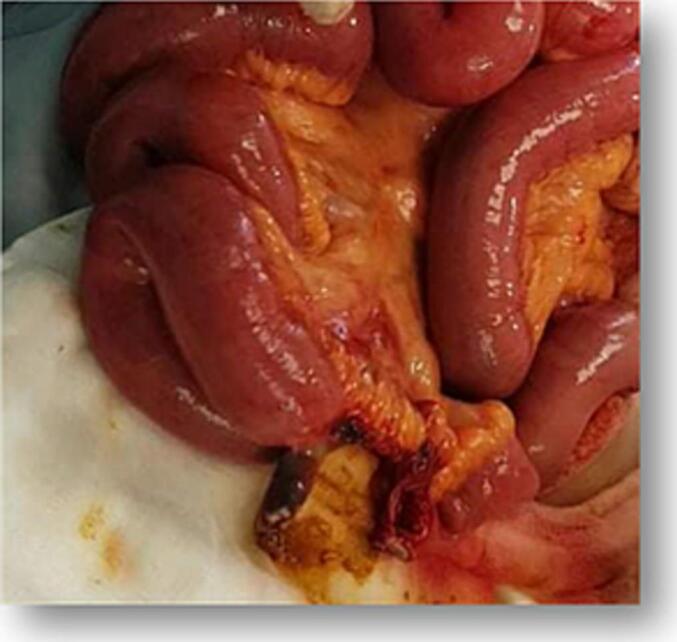
Per Operative views of the inflamed and perforated small bowel.

**Fig. 3:**
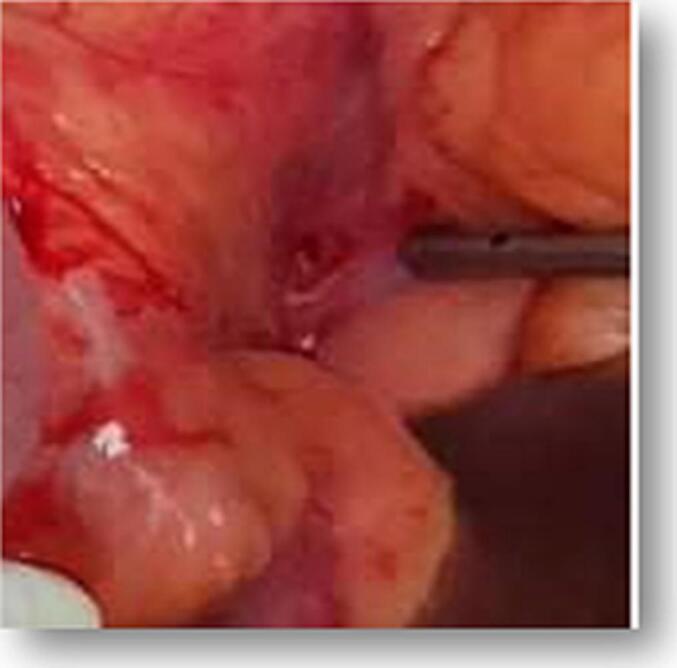
Small hole consistent with the level of L5-S1 disc space on the retro peritoneum.

An urgent laparotomy was performed, revealing a necrotic and perforated ileal segment, located 30 cm from the ileocecal junction, entrapped at the L5-S1 level. A segmental bowel resection with double ileostomy was carried out (Fig. 2). Further inspection of the retroperitoneum identified a small defect at the L5-S1 disc space (Fig. 3), which was subsequently closed.

**Fig. 4:**
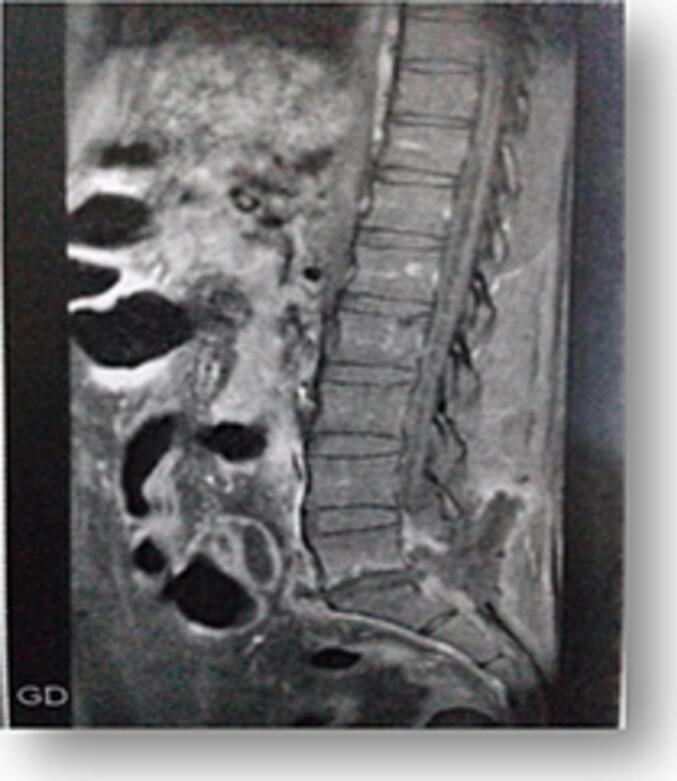
MRI showing lumbar wound collection.

The postoperative course was uneventful except for a lumbar wound infection (Fig. 4) caused by *Klebsiella pneumoniae*, confirmed via wound culture. The infection was successfully managed with appropriate intravenous antibiotics. The patient was discharged 21 days post-surgery.

## 3 Discussion

Intestinal injuries resulting from ventral perforation during lumbar discectomy are exceptionally rare but can lead to severe complications [1]. The anterior portion of the annulus fibrosus is typically thicker than its posterior counterpart and, together with the anterior longitudinal ligament, serves as a robust protective barrier against inadvertent anterior instrument penetration during discectomy. Compared to vascular injuries, isolated bowel injuries following lumbar discectomy occur less frequently. A large-scale study conducted by the German Society of Neurological Surgery reported an incidence of 0.0015 % [9].

The first documented case of intestinal injury in the context of lumbar disc surgery was reported by Harbison in 1954. Subsequently, Schwartz and Brodkey described the first case of intestinal injury specifically following microdiscectomy [5]. To date, a total of 31 cases of bowel injury (Table 1) associated with lumbar disc surgery have been documented in the literature. Twelve publications have specifically detailed cases of bowel injury following lumbar disc surgery or microdiscectomy [1,2,5,8].

**Table 1:** Summary of the cases reported in the literature.

Author	Patient no	Age and Sex	Level of lumbar surgery	Site of intestinal injury	Time to diagnosis	Specifics at diagnosis	Treatment	Outcome	Complication
Harbiso	1	NS*	L4-L5	Ileum	Per operative	Intestinal mucosa found in rongeur	SRA∞	Alive	
DeSaussure	2	NS	NS	NS	NS	NS	NS	NS	NS
DeSaussure	3	NS	NS	NS	NS	NS	NS	NS	NS
Kollbrunner	4	46	L5-S1	Sigmid	7 weeks	Lare bowol obstruction	SRA	Alive	Wound infection
Smith and Estridge	5	52, F	L5-S1	Ileum	2 days	Acute abdomen	PRγ	Alive	Discitis
Birkeland and Taylor	6	30, M	L5-S1	Ileum	1 day	Acute abdomen,	SRA	Alive	
	7	54, M	L5-S1	Appendix	2.5 yrs.	Incidentally discovered at repair of arteriovenous fistula	Appendectomy and fistula repair	Death from gastrointestinal hemorrhage related with previous vessel repair	Discitis
	8	NS,M	L4-L5	Ileum	Peroperative	Intestinal mucosa found in rongeur	PR	Alive	
	9	NS F	L5-S1	Ileum	Several hours	Acute abdomen, fatty tissue found in rongeur	PR	Alive	Wound infection
Shaw et al.	10	35, M	L5-S1	Ileum	3 mos.	Detected by fistulogram	PR	Alive	Discitis with continuous discharge
Shwartz and Brodkey	11	17, F	L4-L5	Sigmoid colon tis	19 days	CT revealed pelvic abscess and free air	PR	Alive	Discis
Smith et al.	12	50, F	L5-S1	Ileum	1 day	Acute abdomen	PR	Alive	
	13	40, M	L5-S1	Ileum	3 days	Acute abdomen	PR	Alive	
Pappas et al.	14	NS	NS	Small intestine	NS	NS	NS	Alive	NS
Goodkin et al.	15	28, F	NS	Jejunum	NS	Acute abdomen	SRA	Alive	
	16	43, F	L5-S1	Small intestine	NS	Acute abdomen	PR	Alive	Discitis
	17	NS	NS	Bowel	Peroperative	A piece of bowel found in rongeur	PR	Alive	
Hoff-Olsen and Wiberg	18	NS, M	L5-S1	Ileum	2 days	Acute abdomen	SRA	Death from abdominal sepsis	
Houten et al.	19	44, F	L5-S1	Jejunum	1 day	Acute abdomen	SRA	Alive	Wound infection
Bilbao et al.	20	36, F	L5-S1	Small intestine	1 day	Acute abdomen	SRA	Alive	
Hollegaard et al.	21	47, M	L5-S1	Ileum	Several hours	Acute abdomen	SRA	Alive	
Shakir and Paterson	22	45,F	L5-S1	Ileum	2 days	Acute abdomen	PR	Alive	
Ebru Sen-Oran	23	30, F	L5-S1	Appendix	3 days n	30, F Acute abdomen	Appendectomy	Alive	Wound infection

NS: Not stated.

SRA: Segmental resection and anastomosis.

PR: Primary repair.

A review of reported cases indicates that bowel injuries most frequently occur during surgery at the L5-S1 level, primarily involving the small intestine, as observed in our case [1]. The timing of injury is a critical factor in establishing an early diagnosis. Clinical presentations can vary significantly, but in most cases, the injury occurs intraoperatively and may be identified immediately if intestinal mucosa is inadvertently excised along with the disc specimen [1,6]. However, in many instances, bowel injury is not recognized until the postoperative period when patients develop signs of an acute abdomen within a few days, as seen in our case. Therefore, postoperative abdominal symptoms should be thoroughly assessed to rule out intestinal injury [4,5].

Abdominopelvic computed tomography (CT) can aid in diagnosis, with approximately one-third of reported cases demonstrating pneumoperitoneum on imaging. Kollbrunner [9,11] described a case presenting with bowel obstruction, in which sigmoid colon stenosis was attributed to local fibrosis and scarring secondary to an organizing retroperitoneal hematoma. In some instances, diagnosis may be delayed by weeks or even months due to the gradual development of a mature intra-abdominal abscess, which initially manifests with mild, nonspecific symptoms [4,8].

Although the prognosis of bowel injury following lumbar discectomy is generally more favorable compared to vascular injury, complications such as generalized peritonitis, septicemia, or concurrent vascular injury resulting from chronic infection can lead to fatal outcomes. Given that delayed diagnosis significantly increases morbidity and mortality, particularly in cases of small bowel perforation, urgent exploratory laparotomy should be performed following prompt diagnostic evaluation. Management strategies for bowel injury vary and may include primary closure of the defect, segmental resection with anastomosis, end-to-end anastomosis with external drainage, or the creation of an ileostomy or colostomy, depending on the severity of the injury and the timing of diagnosis [1,7].

Risk factors for bowel injury can be categorized into two groups: those related to the surgical approach and general predisposing factors. Surgical risk factors include the chosen access route to the disc space, the use of surgical instruments and microscopes, anatomical variations, recurrent disc herniation, and the surgeon's level of experience. General predisposing factors include a history of previous spinal, abdominal, or rectal surgeries, prior radiation therapy, a history of intra-abdominal infections, pelvic inflammatory disease, appendicitis, or diverticulitis, all of which may contribute to the formation of dense adhesions [2,6].

Several precautionary measures may help reduce the risk of this complication. Preoperative imaging should be carefully reviewed to assess the integrity of the anterior annulus fibrosus and anterior longitudinal ligament [8,10]. Additionally, depth marking on surgical instruments is beneficial for estimating the spatial relationship between the instrument and the disc space. As a general guideline, no instrument should be advanced more than 3 cm into the lumbar disc space to minimize the risk of anterior perforation [4,11].

## 4 Conclusion

We report a rare case of intestinal injury following lumbar discectomy. Although this complication is uncommon, it can have severe consequences, potentially leading to generalized peritonitis, septicemia, or chronic infection involving the vertebral column. Proper preoperative planning, incorporating a thorough clinical evaluation and detailed medical history, along with the selection of an optimal surgical approach and meticulous dissection, is essential to prevent ventral perforation and subsequent bowel injury during discectomy. Intestinal injury should be recognized as a potential complication of lumbar disc surgery, particularly in patients presenting with acute abdominal symptoms or persistent wound infections postoperatively. Prompt diagnosis and timely intervention are crucial in preventing life-threatening outcomes.

## Consent

Written informed consent was obtained from the patient for publication of this case report and accompanying images. A copy of the written consent is available for review by the Editor-in-Chief of this journal on request.

## Ethical approval

Ethical approval is exempt/waived at our institution.

Ethics approval is not required for case reports deemed not to constitute research at our institution.

## Funding

This research received no specific grant from the public, commercial, or not-for-profit sectors.

## Author contribution

Hiba Ben Hassine, Faiez Boughanmi, participated in the treatment of the patients and writing the manuscript.

Mohamed Ridha Zayati, Leith limayem, Ibtissem korbi, Faouzi noomen: validation of the manuscript.

All the authors approved the manuscript.

## Guarantor

Hiba Ben Hassine.

## Research registration number

Not applicable.

## Conflict of interest statement

No conflict of interest to disclose. The authors declare no competing interest. The study did not receive any sources of support or funding.
